# The Approach to Sensing the True Fetal Heart Rate for CTG Monitoring: An Evaluation of Effectiveness of Deep Learning with Doppler Ultrasound Signals

**DOI:** 10.3390/bioengineering11070658

**Published:** 2024-06-28

**Authors:** Yuta Hirono, Ikumi Sato, Chiharu Kai, Akifumi Yoshida, Naoki Kodama, Fumikage Uchida, Satoshi Kasai

**Affiliations:** 1 Major in Health and Welfare, Graduate School of Niigata University of Health and Welfare, Niigata 950-3198, Japan; 2 TOITU Co., Ltd., Tokyo 150-0021, Japan; 3 Department of Nursing, Faculty of Nursing, Niigata University of Health and Welfare, Niigata 950-3198, Japan; 4 Department of Radiological Technology, Faculty of Medical Technology, Niigata University of Health and Welfare, Niigata 950-3198, Japan

**Keywords:** Doppler ultrasound, maternal heart rate, fetal heart rate, AI

## Abstract

Cardiotocography (CTG) is widely used to assess fetal well-being. CTG is typically obtained using ultrasound and autocorrelation methods, which extract periodicity from the signal to calculate the heart rate. However, during labor, maternal vessel pulsations can be measured, resulting in the output of the maternal heart rate (MHR). Since the autocorrelation output is displayed as fetal heart rate (FHR), there is a risk that obstetricians may mistakenly evaluate the fetal condition based on MHR, potentially overlooking the necessity for medical intervention. This study proposes a method that utilizes Doppler ultrasound (DUS) signals and artificial intelligence (AI) to determine whether the heart rate obtained by autocorrelation is of fetal origin. We developed a system to simultaneously record DUS signals and CTG and obtained data from 425 cases. The midwife annotated the DUS signals by auditory differentiation, providing data for AI, which included 30,160 data points from the fetal heart and 2160 data points from the maternal vessel. Comparing the classification accuracy of the AI model and a simple mathematical method, the AI model achieved the best performance, with an area under the curve (AUC) of 0.98. Integrating this system into fetal monitoring could provide a new indicator for evaluating CTG quality.

## 1. Introduction

Fetal health assessment is widely performed using cardiotocography (CTG), which simultaneously records fetal heart rate (FHR) and uterine contractions (UCs). Obstetricians assess fetal hypoxia by analyzing the FHR’s baseline, its changes in response to UC, and its variability. Standardized criteria for this assessment have been established by organizations such as the International Federation of Gynecology and Obstetrics (FIGO) as guidelines [1,2]. CTG data are generated through a specialized monitoring system. Due to the consistent pattern of heart activity with each beat, FHR is computed using the autocorrelation coefficient of Doppler ultrasound (DUS), which captures the motion component within the ultrasound signals. The DUS autocorrelation value exhibits peaks corresponding to each heartbeat, and the intervals between these peaks are used to calculate the heart rate. UC is directly derived from readings provided by a pressure sensor [3,4].

The quality of CTG is crucial for obstetricians to accurately assess fetal well-being promptly. A reliable CTG allows for consistent monitoring of UC and changes in FHR. However, situations often arise, particularly during the first and second stages of labor, where CTG quality is inadequate. This includes instances where the onset and intensity of labor contractions are unclear or unreadable and where FHR is frequently lost during monitoring, making diagnosis challenging [5,6,7,8,9].

One reason for signal loss is the effect of artifacts. The algorithm used for autocorrelation to calculate the FHR incorporates all measured DUS signals without distinguishing whether they originate from the fetal heart. If the input signal includes a transient artifact, the correlation coefficient is affected, making it impossible to accurately calculate the heart rate. Another reason is mis-scanning; if the ultrasound beam is not accurately aligned with the fetal heart, the information needed for heart rate calculation is not obtained [3,4].

While FHR is vital for monitoring the well-being of the fetus, signal loss is a common occurrence. It can be due to various factors, such as maternal movement, temporary fetal movements, or the descent of the fetus during delivery. However, signal loss can be promptly addressed, as medical staff, including obstetricians and midwives, can easily detect a decrease in FHR quality by checking whether values are being recorded [5,6,7,8,9]. In addition to visual inspections, automated methods like central systems can inform staff of signal loss [10]. If obstetricians determine that the FHR quality is poor, immediate actions are taken, such as reattaching the monitoring equipment or using an internal measurement method [1,2].

In contrast to signal loss, when the maternal heart rate (MHR) is incorrectly displayed as FHR, a significant delay can occur in taking appropriate action. This is because the displayed heart rate may maintain its continuity; if attention is not paid to the contamination of the MHR, the data will be recognized as of good quality [11]. Failure to differentiate MHR from FHR could result in missed opportunities for medical intervention, potentially impacting fetal outcomes [12].

Midwives and medical staff ascertain the presence of the fetal heart within the scanning area using the emitted DUS sound. Consequently, the MHR is seldom displayed at the commencement of monitoring [1,2,13]. However, in specific scenarios where the fetal heart is displaced from the scanning area owing to maternal movement or fetal shift, and where maternal blood vessels are present within the region, the MHR may appear as the FHR [14]. Determining the MHR based solely on heart rate values is challenging, particularly when the MHR is close to the FHR, making accurate classification difficult [15,16,17]. When the transducer picks up a blood vessel, the medical staff may notice a change in the sound emitted. However, continuous monitoring of sound is impractical owing to its demanding workload. Conventional monitoring systems cannot determine whether the displayed FHR was directly obtained from the fetus. There is also no established methodology that relies solely on the heart rate to confirm the accuracy of the displayed FHR. Therefore, medical practitioners must rely on their assessments by considering the behavior of the heart rate [1,2,11,15,17].

To improve the effectiveness of fetal monitoring, it is crucial to establish a technology that accurately differentiates between FHR and MHR. This advancement is essential and should be rapidly integrated into the clinical practice to enhance patient care. Three approaches have been proposed for addressing this issue. However, each has posed challenges for its widespread use in medical practice or has shown reliability limitations. The first approach reported the potential for enhancement of the obstetricians’ interpretation by eliminating segments where the differences in heart rates between fetal and maternal simultaneous monitoring fell below a threshold [18]. Nonetheless, this method requires additional labor and instrumentation for simultaneous monitoring. The second approach involves the use of electrodes attached to the mother’s abdomen to acquire fetal electrocardiogram (fECG) signals. The fECG system includes a mechanism to remove noise, which minimizes maternal interference [19,20,21,22]. However, this approach requires additional labor and equipment. Because ECG cannot accommodate more than twins, the workflow must be adapted to the number of fetuses, and equipment must be available to monitor more than twins. The third approach employs artificial intelligence (AI). AI indicates whether the heart rate is obtained from the fetus [23]. It uses obstetrician-labeled data from the fetus or mother for training, which can serve as a diagnostic aid for inexperienced obstetricians. However, AI systems cannot handle more critical cases in which obstetricians misidentify MHRs as FHRs.

To facilitate the integration of new technologies into clinical settings, it is crucial to keep existing medical staff workflows uninterrupted. Thus, any adaptation must seamlessly enhance conventional systems to incorporate new functionalities. Furthermore, to improve the accuracy of distinguishing between the FHR and MHR, more informative parameters should be used. The conventional system processes these DUS signals to extract velocity data from the received ultrasonic signals, and subsequently derives cyclical components to calculate the heart rate. However, because heart rate alone is inadequate for differentiating between FHR and MHR, it is necessary to adopt parameters that provide more comprehensive information. The transducers in the system are specifically optimized to capture the target velocity information and the DUS signals were refined within the system.

To address this issue, it is important to utilize DUS signals, which serve as the basis for calculating heart rate. Midwives refer to DUS sounds for positioning when monitoring FHR, and since discernment methods are also described in guidelines, directly utilizing DUS signals may offer the potential to determine whether the fetal heart has been captured [13]. In studies utilizing ultrasonography, it is well-known that characteristic DUS figures can be obtained for each observed blood vessel or organ [24,25,26,27,28,29]. Therefore, there may be differences in the DUS signal between cases in which the fetal heart is captured and those in which it is not. While calculating the displayed heart rate, various pieces of information contained in the DUS signals, such as signal duration, amplitude, and constituent frequencies, are discarded. Additionally, because these data were not recorded by a device, research in this area has not yet progressed. We selected AI as the classification method for the DUS signals because of its well-established efficacy for sound [30,31].

Hence, our objective was to propose a methodology for assessing the quality of FHR using DUS signals as indicators. To determine whether the FHR observed on CTG originated from the fetal heart or was indicative of maternal heartbeats, we developed an AI system capable of categorizing the source of the DUS signals used as input.

## 2. Materials and Methods

### 2.1. Data Acquisition

The conventional system was modified to transmit the DUS signal and CTG information via Ethernet to the central system because the sound generated by the monitoring equipment was not recorded. In cases of simultaneous maternal monitoring, the system also transmitted MHR through Ethernet. The data were obtained from a single facility, processed by TOITU Ltd. to remove personal information, and then provided for this research.

The data collection period spanned from June 2022 to August 2023. The participants were pregnant women aged 20 years and older, with gestational ages ranging from 24 to 42 weeks. No cases of twins or more, fetal deaths, or maternal deaths were included. Measurements were taken during the first and second stages of labor. The CTG monitors utilized in this study were the MT-610 and MT-516 models (TOITU Ltd., Tokyo, Japan). The FHR, UC, and MHR were sampled at a frequency of 4 Hz. Concurrently, DUS signals were recorded at a frequency of 1 kHz with a 16-bit resolution using custom software. A total of 425 recordings were made, resulting in a total recording time of 4,147,203 s.

### 2.2. Labeling DUS Signals

**Figure 1:**
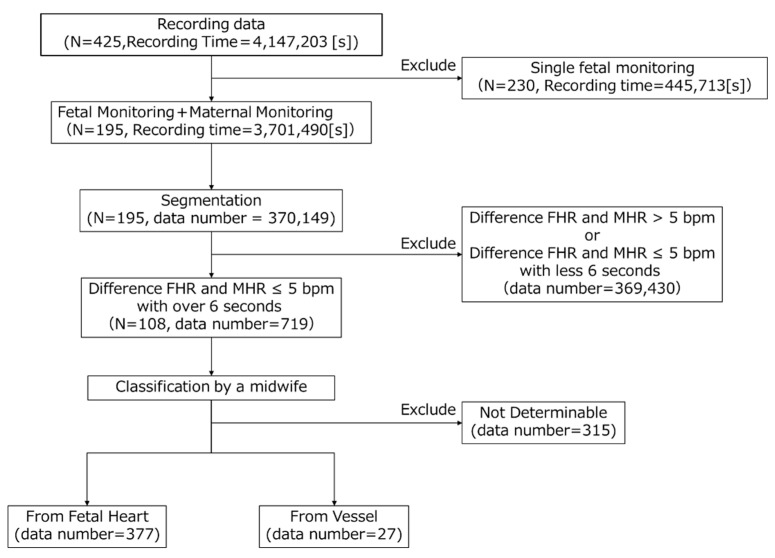
Flowchart of labeling data.

To create the AI dataset, the DUS signals were labeled by a midwife with clinical experience. To ensure efficiency and classification accuracy, we consulted with the midwife and conducted the process in four steps. Figure 1 in the paper illustrates the flowchart depicting the labeling process by midwives on the measurement data. In the first step, we extracted cases from the dataset where FHR and MHR were recorded simultaneously. We focused solely on simultaneous recordings because MHR was to be used as auxiliary data in subsequent processes. There were 195 such recordings, totaling 3,701,490 s. In the second step, data for the midwives to label were created, and the FHR, MHR, and DUS signals were divided every 10 s. The data segments were kept short to prevent mixing multiple categories and ensure they were of a length suitable for midwives to identify accurately. In total, 370,149 segments were created. In the third step, the data extracted in the second step were further selected based on the difference between the FHR and MHR. Segments where the difference was less than 5 bpm for at least 6 s were selected. This condition was adopted to efficiently obtain two categories of DUS signals, as the displayed FHR could be MHR [18]. This resulted in 719 segments within the 108 recordings. In the fourth step, the midwife categorized the DUS signals into “From Fetal heart”, “From Vessel”, and “Not determinable” by listening. The plotted DUS signal was also shown as an auxiliary to indicate the signal switching. The results showed that 377 segments were classified as “From Fetal Heart”, 27 segments as “From Vessel”, and 315 segments as “Not Determinable”. The “From Fetal Heart” category was extracted from 74 records, and the “From Vessel” category from 7 records. In four of these records, both the “From Fetal Heart” and “From Vessel” categories were obtained. Additionally, cases that included artifacts such as fetal movements, those with a mix of “From Fetal Heart” and “From Vessel”, and those where the amplitude of the DUS signals was too low to be audible were categorized as “Not Determinable”.

### 2.3. Datasets for AI

To conduct hold-out validation, we prepared data for AI training and divided it into training, validation, and test sets with a ratio of 23:2:2. This ratio was based on the number of segments in the “From Vessel” category. Midwife-labeled 10 s segments were assigned to each dataset. Initially, for each category, two 10 s segments for “From Vessel” and 28 for “From Fetal Heart” were randomly allocated as test data. The remaining segments were reserved for training and validation purposes.

To ensure sufficient data volume for effective AI learning, we augmented the segmented data [32,33]. Specifically, we set the length of the data used by the AI to 2 s and extracted data sequentially from 10 s segments. The extraction positions were shifted every 100 ms based on the starting position of the segment. This allowed us to extract 80 data points for AI use from each segment. In this study, we set the data length to 2 s regardless of the recorded heart rate on the CTG to ensure that one beat is always included. The data length was calculated based on the lowest heart rate displayed by the device, which was 50 bpm, serving as the standard. Setting an appropriate length was crucial because if the data length is too long, multiple signals may mix, making it difficult for AI to make judgments.

Table 1 displays the augmented data points for each label, with 30,160 instances labeled “From Fetal Heart” and 2160 instances labeled “From Vessel”. Each label was split in a 23:2:2 ratio for training, validation, and testing. The training and validation data for the “From Vessel” label were expanded by a factor of 14 due to imbalances in the amount of data between labels. Once the data augmentation was completed, the data were divided randomly such that 92% was for training data and 8% for validation data. Table 2 shows the datasets for the AI performing holdout validation.

### 2.4. AI Model and Performance Evaluation

**Figure 2:**
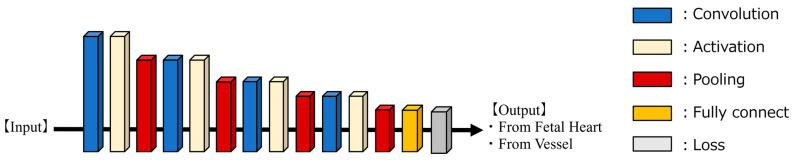
AI-model architecture for 1D-CNN.

As illustrated in Figure 2, we developed a supervised learning AI model tailored for binary classification using 1D-CNN. The core architecture consisted of a sequence of convolutional layers, activation layers (ReLU), and MaxPooling layers, interconnected and repeated four times. Following this sequence, the SoftMax function was employed, and the cross-entropy method was used as the loss function. The probability for each class was calculated, and the class with the highest probability was assigned. The model was trained for 100 epochs, with a batch size of 64 and a learning rate of 6.066 × 10^−4^. The learning rate was determined using Optuna, an optimization software, to minimize losses [34]. The computational infrastructure utilized for both training and evaluation included an Intel(R) Core (TM) i9 series CPU operating at 3.00 GHz and an NVIDIA GeForce RTX 3090 GPU. The experiments were conducted using Python (version 3.9.16; Python Software Foundation, Wilmington, DE, USA) with the PyTorch framework (version 2.1.1).

When evaluating the performance of a model, accuracy is often the most straightforward metric. However, in cases of unbalanced data, accuracy alone may not provide a reliable evaluation. Therefore, we also included additional metrics such as precision, recall, and F1-score to comprehensively evaluate the model’s performance. These metrics help in understanding how well the model performs across different aspects of classification.

The results are categorized into four groups: true negatives (TNs), false negatives (FNs), true positives (TPs), and false positives (FPs).

Accuracy measures the proportion of correctly predicted samples across the entire dataset, providing an overview of the overall performance of the model.

(1) $$Accuracy=\frac{TP+TN}{TP+FP+TN+FN}\times 100\;[\%]$$

Precision indicates the proportion of samples predicted as positive by the model that are truly positive, providing insights into the impact of false positives.

(2) $$Precision=\frac{TP}{TP+FP}\times 100\;[\%]$$

Recall quantifies the proportion of samples correctly predicted as positive by the model out of all actual positive samples, providing insights into the impact of false negatives.

(3) $$Recall=\frac{TP}{TP+FN}\times 100\;[\%]$$

The F1-score is a harmonic mean of precision and recall, offering a balanced assessment of the model’s performance across both metrics.

(4) $$F1\text{-}Score=\frac{2\times Precision\times Recall}{Precision+Recall}\times 100\;[\%]$$

We evaluated whether the classification accuracy of the AI model was superior to that achieved through simple signal processing. To confirm this, the Receiver Operating Characteristic (ROC) curves were generated for each metric, and the Area Under the Curve (AUC) was computed. The ROC curve illustrates the diagnostic ability of a binary classifier system across various threshold settings, plotting the true-positive rate against the false-positive rate. The AUC serves as a measure of the overall performance of the classifier, with a higher AUC indicating better discrimination between positive and negative cases.

Simple signal processing techniques were used to calculate the following four metrics, which were then utilized to derive the ROC curves and AUC for the test data. These metrics were also compared against the results from an AI model. After taking the absolute values of the data, the maximum value was computed as “Peak Amplitude”. Similarly, the average value after taking the absolute values was calculated as “Average Amplitude”. Fast Fourier Transform (FFT) was conducted, where the fundamental frequency was computed from the resultant frequency characteristics, and its power was defined as “Central Frequency Intensity”. The frequency width representing half the power of “Central Frequency Intensity” was calculated as “Half-power Band Width”. ROC curves and AUC were computed for the test data using these metrics, and comparisons were made with those derived from the AI model to evaluate its performance.

## 3. Results

The AI model trained on the training and validation datasets, as shown in Table 2, was utilized to evaluate the test dataset, resulting in the confusion matrix presented in Table 3. TP represents the conditions where both ground truth and AI-predicted labels are “From Fetal Heart”, while TN denotes cases where both are “From Vessel”. FP indicates combinations in which the true label is “From Vessel”, the AI-predicted label is “From Fetal Heart”, and FN represents the reverse combinations of FP. The results presented in Table 3 indicate that the calculated accuracy, precision, recall, and F1-score were 97.9%, 82.3%, 86.9%, and 84.5%, respectively.

**Figure 3:**
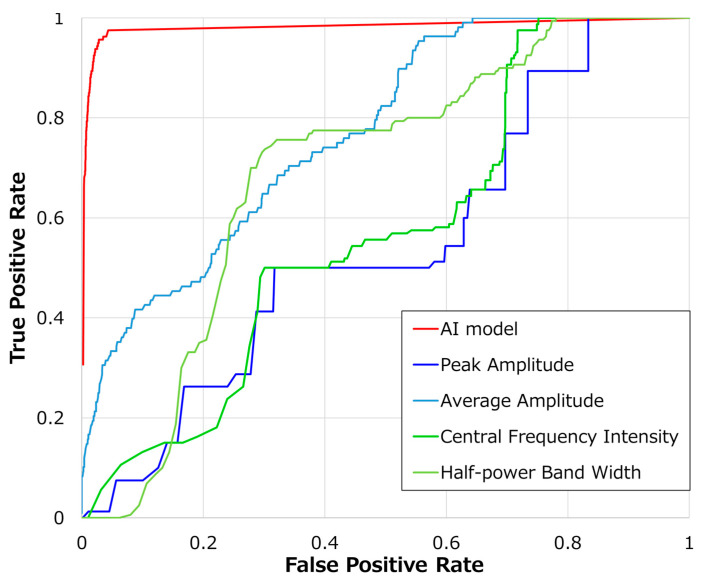
Comparison of AI model and threshold-based ROC curves.

In Figure 3, we compared the classification accuracy of our AI model with classical signal analysis methods using ROC curves. The red line represents the results of the AI model proposed in this study, the blue line shows maximum amplitude, light blue represents average amplitude, green represents fundamental frequency, and yellow-green depicts half-width frequency. Table 4 presents the AUC values for each method. Our AI model achieved the highest AUC of 0.98, with the average amplitude being the highest among classical signal analysis methods. Conversely, central frequency intensity had the lowest value at 0.50.

## 4. Discussion

This study is significant because it showcases the ability to evaluate FHR quality using DUS signals. The FIGO guidelines have delineated methods for distinguishing DUS sounds between vascular and fetal heart origins [13]. Moreover, since DUS signals during fetal monitoring reflect cardiac function, there has been a suggestion that differentiating DUS signals from blood vessels and those from the fetal heart observed through ultrasonography might be feasible [3,4,24,25,26,27,28,29]. Additionally, the advantage of classifying using DUS signals lies in not disrupting the workflow of medical staff, as no additional equipment is needed. However, to the best of our knowledge, no prior study has developed a system to differentiate these signals using DUS. One possible reason for this gap is the similarity between signals from the fetal heart and maternal blood vessels. As shown in Table 4 and Figure 3, achieving satisfactory accuracy with simple signal analysis methods is difficult. This study demonstrates that employing AI can lead to high classification accuracy. Another factor could be that existing systems only output DUS sounds through speakers without preserving them. To conduct a thorough analysis, it was crucial to develop a system capable of collecting DUS signals, as demonstrated in this study, which may elucidate why this area has not been extensively explored before.

In this study, the developed AI model achieved an accuracy of 97.9%. In other studies that used the UrbanSound8K dataset for audio-acoustic analysis with CNN, accuracies ranged from 89.3% to 94.5%. Despite the differences in model configuration and tasks, the results of this study are deemed appropriate for the accuracy of an audio classification task [30]. In addition, in examples where AI models were used for detecting the inclusion of MHR in FHR analysis, the accuracy was reported to be 99.93%, suggesting that the performance of the studied approach is nearly equivalent [23]. The determination of whether analyses using FHR or DUS signals are more suitable for detecting MHR can be clarified by future comparative studies using the same data.

As shown in Table 3, the AI demonstrated the capability to distinguish fetal-origin DUS signals. The rationale for classifying DUS signals stems from the understanding that each measurement site exhibits unique characteristics in its signals [29]. Notably, differences in shape exist between signals with multiple amplitude peaks per beat, such as those from the heart, and signals with only one amplitude peak per beat, such as those from blood vessels [3,24,27,28,29]. However, as depicted in Figure 3 and Table 4, basic signal analysis lacked the accuracy needed for classification, thus posing challenges for automation. The limitation in making frequency-based distinctions was attributed to the narrow-band filtering applied to DUS signals to bring them within the audible range. This filtering did not generate adequate frequency feature differences to enable clear differentiation between the categories [3]. Furthermore, the difficulty in classifying based on amplitude may stem from dynamic amplitude variations based on measurement conditions, making it difficult to establish a consistent criterion. These observations led us to conclude that AI is essential for effectively classifying DUS signals based on their features.

**Figure 4:**
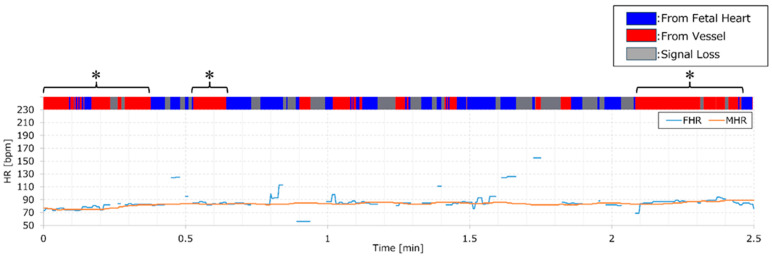
Color bar of AI output and FHR/MHR displayed in parallel. An illustration of simultaneous monitoring values and AI determination results, with FHR denoted by a blue line and MHR by an orange line. At the top of the graph, a color bar indicates AI output results: fetal source displayed in blue and vessel source in red.

The potential applications of the AI model developed in this study are noteworthy. For example, integrating AI into medical devices can trigger alarms and display FHR on the screen, indicating the reliability of the heart rate measurement. Figure 4 provides an example where monitoring values and AI judgment results are displayed simultaneously. It should be noted that these results encompass data used for both training and validation. However, an increase in the red section of the color bar may signify missed fetal heartbeats, indicating a decline in monitoring quality. The solid orange and blue lines in Figure 4 represent MHR and FHR, respectively. The asterisks highlight regions with a higher proportion of red, as determined by an AI analysis of the DUS signal. These regions align with intervals where MHR and FHR are close, underscoring the degradation in FHR quality when an MHR monitor is not utilized. Identifying such intervals when solely monitoring FHR requires specialized knowledge. However, employing this system makes it simple to identify deteriorated quality segments without simultaneous MHR measurement by observing the upper color bar. This could prompt actions like device repositioning or conducting thorough examinations when quality degradation is noted.

**Figure 5:**
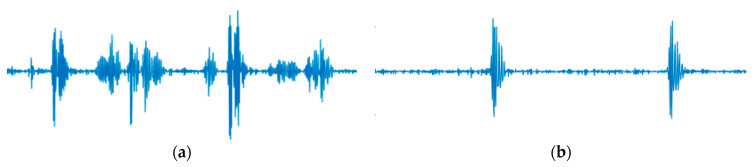
Examples of DUS signals are classified by the midwife. (**a**) Example of “From Fetal heart” (**b**) Example of “From vessel”. The signal lengths of (**a**,**b**) are both 1 s. Both examples record 2 beats.

There are two types of limitations identified in this study: one pertains to the AI model, and the other relates to the truthing process. While this study concentrated on singleton pregnancies, future research should encompass data from multiple pregnancies, which often present more artifacts from fetal movements that could potentially degrade DUS signal quality. Real-time processing is imperative for clinical use; although we employed offline analysis here, verifying real-time feasibility in a central system is essential for clinical applicability. Furthermore, this approach necessitates further clinical evaluation; depending on the outcomes, enhancements in accuracy may be required. Several methods are available to improve AI accuracy, such as leveraging advanced AI models like Transformer and ResNet [35,36], and/or incorporating semi-supervised learning techniques with “not determinable” data [37,38]. A limitation of this study lies in the reliance on labeling by a single midwife. We assumed that a single healthcare provider would handle device installation in typical fetal monitoring scenarios, anticipating accurate classification even with one person’s labeling. Upon reviewing the midwife’s criteria, “From Fetal Heart” was identified using the galloping horse criterion, while “From Vessel” was based on one pulse per beat, aligning with FIGO standards concerning sound quality [13]. Figure 5 visually represents this midwife’s classification, illustrating the DUS waveform.

## 5. Conclusions

This study proposed an AI system utilizing DUS signals and validated its capacity to accurately identify fetal heart-derived signals. The DUS signal may contain information related to the motion of the observed subject and is suitable for the identification of the measurement targets. Identifying MHR from continuously recorded FHR was challenging; however, incorporating this system into fetal monitoring opens avenues for healthcare staff to access novel indicators for evaluating CTG quality.

## Figures and Tables

**Table 1:** Number of labeled data points.

Data Label	Number of Data Points
From Fetal Heart	30,160
From Vessel	2160

**Table 2:** Number of data points in each dataset for AI.

Data Label	Number of Data Points		
	Training	Validation	Test
From Fetal Heart	25,668	2252	2240
From Vessel	25,778	2222	160

**Table 3:** Confusion matrix of AI output.

		AI-Predicted Labels	
		From Fetal Heart	From Vessel
True labels	From Fetal Heart	2210	30
	From Vessel	21	139

**Table 4:** The performance of the AI model and each threshold-based parameter.

Parameter	AUC
AI Model	0.98
Peak Amplitude	0.54
Average Amplitude	0.76
Central Frequency Intensity	0.50
Half-power Band Width	0.69

## Data Availability

The data used in this study are available upon request from the corresponding author.
